# Insulin-Like Growth Factor-1 and Selected Insulin-Like Growth Factor Binding Protein Concentrations during an Ultramarathon Sled Dog Race

**DOI:** 10.1155/2016/5686372

**Published:** 2016-09-05

**Authors:** Matthew W. Brunke, Christopher W. Frye, Corri B. Levine, Cristina Hansen, Joseph J. Wakshlag

**Affiliations:** ^1^North Country Veterinary Referral Center, 454 Queensbury Avenue, Queensbury, NY 12804, USA; ^2^Department of Clinical Sciences, College of Veterinary Medicine, Cornell University, Tower Road, Ithaca, NY 14853, USA; ^3^Department of Biology and Wildlife, The University of Alaska Fairbanks, Fairbanks, AK 99775, USA

## Abstract

The objective of this study was to investigate the effects of running a 1000-mile (1600 km) endurance sled dog race on serum insulin-like growth factor-1 (IGF-1) and insulin-like growth factor binding proteins 1 and 3 (IGFBP-1 and IGFBP-3). Serum was examined from 12 sled dogs prior to the race, at midrace (approximately 690 km), and again at the finish. IGF-1, IGFBP-1, and IGFBP-3 were assessed using radioimmunoassay or enzyme linked immune-absorbance assays. Mean prerace concentrations were significantly higher than midrace and end-race concentrations at 215.93 ± 80.51 ng/mL, 54.29 ± 25.45 ng/mL, and 55.53 ± 28.25 ng/mL, respectively (*P* < 0.001). Mean IGFBP-1 concentrations were not different across these time periods at 24.1 ± 15.8 ng/mL, 25.7 ± 14.0 ng/mL, and 26.6 ± 17.6 ng/mL, respectively. IGFBP-3 concentrations showed a modest significant decrease across time periods at 3,067 ± 2,792 ng/mL, 2,626 ± 2,310 ng/mL, and 2,331 ± 2,301 ng/mL, respectively (*P* < 0.01). Endurance sled dogs show a precipitous drop in serum IGF-1 concentrations. These differences may be related to fuel utilization and excessive negative energy balance associated with the loss of body condition during racing. The relative stability of IGFBP-1 and IGFBP-3 suggests that IGF-1 anabolic signaling is diminished during ultramarathon racing. Further studies comparing the influence of time and duration of exercise versus negative energy balance on serum IGF-1 status are warranted to better understand exercise versus negative energy balance differences.

## 1. Introduction

Insulin-like growth factor-1 (IGF-1, somatomedin C) is an anabolic peptide hormone similar in molecular structure to insulin with some insulin-like actions. Growth hormone (GH) stimulates IGF-1 production and release. As IGF-1 concentrations increase in the blood stream, they provide a negative feedback loop upon GH release in the pituitary gland. The primary site of action of IGF-1 is insulin-like growth factor-1 receptor (IGF1R). This cellular receptor is found throughout the body and helps drive anabolic protein synthesis and glucose uptake and utilization similar to insulin receptor actions. Such actions are reflective of IGF-1 acting as a potent stimulator of cell growth, proliferation, and hypertrophy [1].

While IGF-1 is within the bloodstream, 99% is bound to insulin growth factor binding proteins (IGFBPs) [2].

Six IGFBPs have been identified, cloned, and sequenced. These molecules interact with IGF-1 in the serum and all have a higher affinity than the IGF-1 cell receptors and may limit available ligand for biological signaling. Additionally these proteins differ in their structure, and other functions have been proposed including transportation, prolonging IGF half-lives, providing tissue or cell specificity, or even neutralizing and potentiating IGFs [2–5]. IGFBP-3 is the most abundant of the IGFBPs and accounts for 80% of all IGF binding.

IGFBPs 1 and 3 have been examined in human athletes undergoing short bouts of exercise, demonstrating no change over 30 minutes [6]. In marathon runners IGFBP-1 concentrations were elevated just after the race and returned to normal in 24 hours [7]. Human studies showed no immediate change in IGFBP-3 after marathon running, but mild increases may occur 1 to 3 days after the marathon when plasma volume changes are considered [7].

Conversely, concentration differences in IGF-1 have been noted across different types of exercise. Baseline IGF-1 will be higher in well-trained athletes as compared to sedentary people. While individual serum IGF-1 concentrations appear to remain the same during the course of a marathon, sprinting activities have shown increases [7, 8]. Additionally, endurance athletes running over multiple days have demonstrated decreases in IGF-1 concentrations [9].

Currently there is limited information regarding canine IGF-1 and associated binding proteins [10] and no studies examining the effects of exercise on IGF-1 and IGFBPs in dogs, particularly those undergoing endurance exercise. Given the paucity of information available, we elected to study changes in serum concentrations of IGF-1 and selected IGFBPs within a group of competitive ultramarathon racing sled dogs during the course of the entire 2015 Yukon Quest 1,000-mile International Sled Dog Race.

## 2. Materials and Methods

### 2.1. Dogs

Dogs were recruited from 3 teams to participate in the study after providing informed client consent. The study protocol was approved by the Cornell University Institutional Animal Use Committee and the Yukon Quest Board of Directors.

Venipuncture was conducted on 14 dogs from each team approximately 24 hours prior to the start of the race in Whitehorse, Yukon Territory, with plans for further collections at the midway point at Dawson City, Yukon Territory (approximately 480 miles into the race), and then again at the finish in Fairbanks, Alaska (approximately 983 miles). All blood samples were taken within 2 hours of the dogs stopping at the midway point in Dawson City and at the finish in Fairbanks, Alaska. Of the original teams bled, only one team had follow-up blood draws. Fourteen dogs (3 were female and 11 were male) were bled at the midpoint of the race in Dawson; 2 dogs were discontinued from the race between the midway point and the finish leaving only 12 dogs for blood collection in Fairbanks.

### 2.2. Sample and Data Collection

Ten mL of whole blood was collected using a 22-gauge needle and 12 cc syringe from each dog at the respective time points. The blood was immediately transferred to a coagulation tube and allowed to clot and stored on ice for approximately 30 minutes before centrifugation at 4,000 ×g for 10 minutes. Serum was then stored in 1 mL aliquots and kept frozen on dry ice for transportation to the principal investigator's lab approximately 15 days later. Once received the samples were stored at −80°C until thawed for assays. All dogs had body condition scores evaluated at the time of all blood draws according to the 1–9 scoring system [11] to determine if the dogs changed body condition during the race.

### 2.3. RIA and ELISA Procedures

The IGF-1 concentrations in each serum sample were assayed using an immunoradiometric commercial kit based on two region-restricted affinities purified polyclonal antibodies (IGF-1: IGFR22 Diasorin, Mediagnost, Reutlingen, Germany) [12] and was used according to the manufacturer's instructions. Intra-assay and interassay coefficients of variation were 5.2% and 21.3%, respectively, using the standard additions method for evaluation of precision and linearity, while a low and high canine IGF-1 standard (E90050Ca, Cloud-Clone, Houston, TX, USA) were a control for accuracy.

Canine IGFBP-1 was measured via enzyme linked immunoabsorbance assay (ELISA; canine insulin-like growth factor; MB022518, MyBioSource, San Diego, CA, USA).

The kit was used according to the manufacturer's instruction using undiluted whole serum which provided values within the standard curve of the assay. High and low limits of detection were 15.6 to 500 ng/mL with inter- and intra-assay coefficients of variation determined by the manufacturer of less than 15% for both.

Canine IGFBP-3 was measured using ELISA (canine insulin-like growth factor; MB022518, MyBioSource, San Diego, CA, USA). The kit was used according the manufacturer's instructions with undiluted whole serum to provide values within the standard curve of the assay. If a value was found to be above the high limit of detection, the sample was diluted 1 : 2 to provide a value within the standard curve generated in the assay (3 samples required dilution). High and low limits of detection were 100 to 6,000 ng/mL with inter- and intra-assay coefficients of variation determined by the manufacturer of less than 10% for both. All samples from a single dog across time points were run on the same plate for both ELISAs. For both IGFBP-1 and IGFBP-3 samples were run in duplicate and if the duplicate values have over a 15% disparity the sample was repeated.

### 2.4. Statistical Analysis

Results from each assay were assessed for normality using a Shapiro-Wilk test with IGF-1 and IGFBP-1 proving to be normally distributed data, while the IGFBP-3 data proved to be skewed. IGF-1 and IGFBP-1 were statistically evaluated using a mixed model repeated measured analysis of variance, while IGFBP-3 was assessed using a mixed model Friedman's test. All tests underwent Tukey's post hoc analysis to determine if there were any significant differences between preracing samples, midrace, and finishing values for each group. A *P* value of 0.05 was set as significance for all statistics analyses.

## 3. Results

### 3.1. Dogs and Body Condition Scores

All 14 dogs that started the race completed running to the midway point and one dog was dropped from the race at this juncture in Dawson City. Approximately 160 kilometers further another dog was dropped from the race leaving 12 dogs that finished whose serum was evaluated at all three time points and are reported. The median and range body condition score (BCS) at the start of the race was 5.0 (range 4-5). Midrace at Dawson the median BCS was 4.0 (4.5–3.5) and the median BCS was 3.0 (3.0–3.5) at the race finish. Dog name, age, gender, and starting and ending body conditions can be found in Table 1.

### 3.2. IGF-1 Serum Concentrations

**Figure 1:**
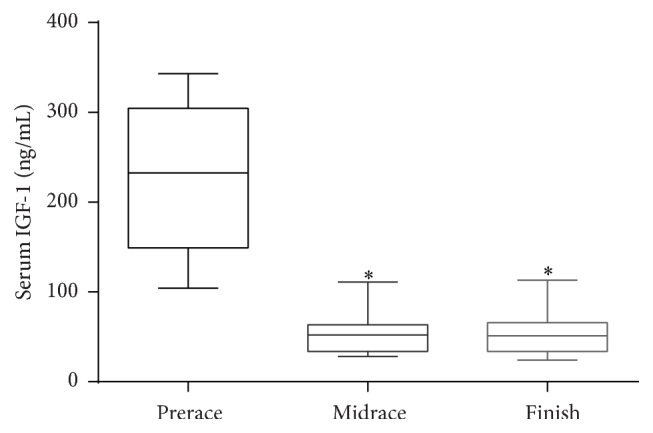
IGF-1 mean and standard deviation at prerace, midrace, and race finish. Boxes represent mean and 75th and 25th percentiles and whiskers represent 1st and 99th percentiles. *∗* indicates a significant difference from the prerace values (*P* < 0.01).

IGF-1 concentrations decreased significantly between prerace and midrace in Dawson City. The mean prerace concentration was 215.93 ± 80.5 ng/mL and the mean halfway point concentration was 54.29 ± 25.5 ng/mL (*P* < 0.01). The mean IGF-1 serum concentration at the termination of the race was 55.33 ± 28.3 ng/mL, which was significantly different from the preracing concentrations (*P* < 0.01). There was no significant difference between the midrace and race finish concentrations (Figure 1).

### 3.3. IGFBP-1 and IGFBP-3 Concentrations

**Figure 2:**
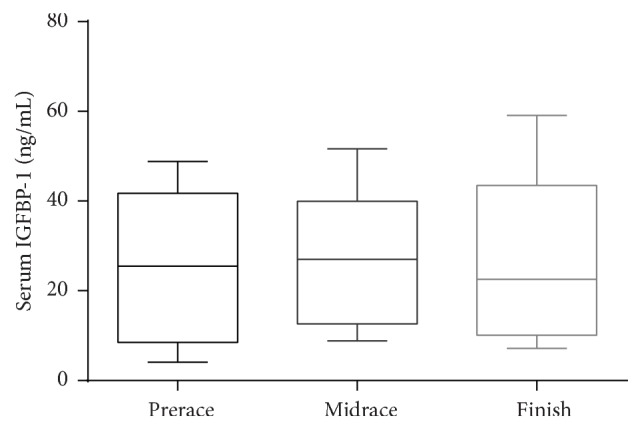
IGFBP-1 at prerace, midrace, and race finish. Boxes represent mean and 75th and 25th percentiles and whiskers represent 1st and 99th percentiles.

**Figure 3:**
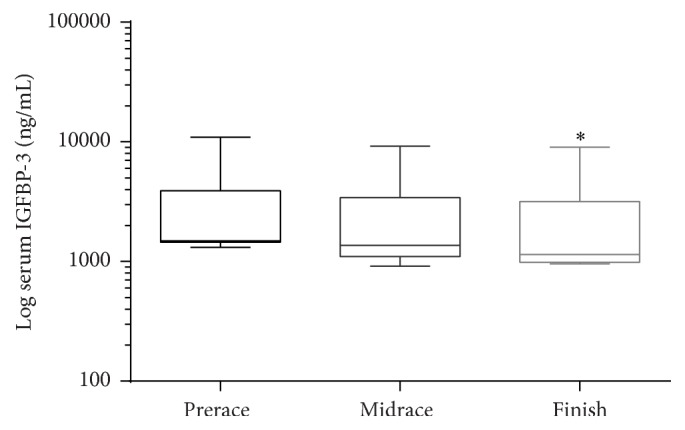
IGFBP-3 at prerace, midrace, and race finish. Boxes represent median and 75th and 25th percentiles and whiskers represent 1st and 99th percentiles. *∗* indicates a significant difference from the prerace values (*P* < 0.01).

Mean serum IGFBP-1 concentrations at prerace were 23.1 ± 15.8 ng/mL. By midrace the IGFBP-1 concentrations were 25.7 ± 14.0 ng/mL, which was not significantly different from the preracing mean. At completion of the race the serum IGFBP-1 concentrations were 26.6 ± 17.6 ng/mL showing no significant difference from prerace or midrace concentrations. (Figure 2). Serum IGFBP-3 concentrations at prerace were 3,067 ± 2,792 ng/mL and were not significantly different from midrace concentrations, 2,626 ± 2,310 ng/mL, while race finish concentrations were 2,331 ± 2301 ng/mL and were significantly different from preracing values (*P* < 0.01), but not midrace concentrations (Figure 3).

## 4. Discussion

The IGF-1 concentrations in racing sled dogs responded unexpectedly with a dramatic significant decrease during exercise. Interestingly, no alterations in the IGFBP-1 concentrations were observed. IGFBP-3 in racing sled dogs responded similarly to that of human marathon runners at the halfway point with no significant decrease; however by day 10 of racing IGFBP-3 did decrease significantly compared to prerace values [7]. Most studies of human athletes [13, 14] have shown mild increases in IGF-1 and IGFBP-1 during prolonged exercise such as marathon. It has been suggested that IGFBP-1 has a role in glucose counter regulation by binding and delivering IGF-1 to tissues maintaining glucose homeostasis in people, particularly muscle tissue [15]. In racing sled dogs the precipitous drop in IGF-1 and relatively low IGFBP-1 suggest other mechanisms are important in delivery of glucose to muscle tissue. IGF-1 concentrations in people will decrease with prolonged exercise over a 7-day period, but not an 80% drop like that experienced in this group of endurance sled dogs [16]. Smith and colleagues found that the decrease in IGF-1 with sustained exercise is similar to that of the decrease of IGF-1 with caloric restriction [16]. In each case the IGF-1 concentrations dropped by approximately 25%, reflecting the extent of negative caloric balance incurred by long duration strenuous exercise in normal individuals. Similarly, IGF-1 and IGFBP-3 decreased approximately 25% and 20%, respectively, in people over a 100-kilometer human marathon race. Participants in this race had access to food and water as needed, suggesting an exercise component to the decreases of serum concentrations [17]. However, a total calorie deficit may have persisted despite access to food. The intensity of exercise is much greater in our racing dogs comparatively as seen by the loss in body condition during the race. This negative energy balance without time for the body to recover is the most likely cause for the dramatic decrease in serum IGF-1 observed in these racing dogs.

The precipitous weight loss observed, which is also observed in human athletes to a lesser degree [18], shows severe negative energy balance in these endurance dogs. Body condition scoring suggests a 7–10% decrease in weight with each one point drop in BCS; therefore the dogs in our study lost a mean 14–20% of their individual body weight during the course of the race [11]. Previous studies by Hinchcliff and colleagues have shown that sled dogs expend approximately 11,250 kilocalories a day and metabolizable energy intake was approximately 10,600 kilocalories per day during a 70-hour race [18]. Another study showed that sled dogs were typically fed 9,500 to 12,000 calories per dog per day and still lost weight (5–10%) throughout the course of the Yukon Quest Ultramarathon sled dog race [19]. Unfortunately, there were logistical problems during the race that precluded collection of body weights and although kilocalories intake was not assessed in this study the feeding pattern of this kennel during racing was examined in a prior study [19].

The precipitous drop in IGF-1 (approximately 80%) is similar to a study where dogs were reduced to approximately 60% of their normal metabolic energy requirement and consequently experienced weight loss [10]. Although IGF-1 changed in our racing dogs, the selected binding proteins were not altered dramatically. On the other hand, a caloric reduction in cats led to downregulation of IGF-1 synthesis by 51% with only a 42.5% restriction in calories, without alteration in the IGFBP-1 or IGFBP-3 from a comparative perspective [20]. Because prolonged exercise in people leads to changes associated with negative energy balance [16] and calorie restriction in companion animals results in decreases of IGF-1 concentrations, it seems intuitive that the IGF-1 and BCS changes seen in our study dogs reflect a prolonged and extreme negative energy balance that is reflected by the largest decrease in IGF-1 ever observed in a canine or human model of exercise.

Little alteration in IGFBPs was observed in this study. IGFBP-1 concentrations did not significantly change during the course of the race, while IGFBP-3 did show a small, but statistically significant decrease by the end of the race. The changes in IGFBP-3 were negligible when compared to the decreases in IGF-1. There have been mixed results with IGFBP-1 concentrations in people pending the duration and type of exercise. Over an 8-week period of concentric training in people there is a decrease in IGFBP-1, while eccentric exercise in humans caused mild increases [21]. In day-long exercise (eccentric or concentric) no significant changes were seen in IGFBP-1 concentrations in people [22]. Marathon runners [7] exhibited modest increases in IGFBP-1. In people, marathon racing and heavy resistance exercise bring about equivocal changes in IGFBP-3 concentrations [7, 22, 23].

Concentrations of IGFBP-3 in these dogs were similar to human athletes and did significantly decrease by the end of racing. However this change, as with IGF-1, may not be a direct reflection of prolonged racing and exercise, but possibly a marker of the energy expenditure leading to overall decreased hepatic synthesis of these binding proteins due to metabolic demands of racing for 9-10 consecutive days.

There are limitations of our study when discussing the dynamics of IGF-1 and IGFBPs during exercise as we did not collect serum until day 4 of racing at midrace; therefore we cannot comment on possible transient IGF-1 or binding protein changes that might occur due to exercise, but the lack of increase over the entire race suggests that exercise plays little to no role in long term homeostasis of IGF-1 in the face of ultramarathon endurance exercise. Lastly, we could not separate the effects of negative energy balance versus exercise on the IGF and IGFBPs we measured but expect that with the loss in body condition that negative energy balance plays a more significant role considering the extent of IGF-1 decreases observed.

## 5. Conclusion

IGF-1 concentrations decrease similarly to people performing long duration exercise (greater than 24 hours), but to a far greater extent. However, negative energy balance may be playing a significant role (as opposed to exercise directly) over the influence of IGF-1 concentrations in our ultramarathon racers. This same energy balance may also explain the decrease in IGFBP-3 during the course of the race as it is impossible to separate the effects of exercise from that of the energy balance. Examination at earlier time points and different cohorts of exercising canines may shed light into the severity of IGF-1 downregulation due to exercise versus energy intake/expenditure imbalance, providing a better understanding of the influence of exercise on IGF-1 concentrations. The tremendous decrease in IGF-1 sheds light regarding the extreme negative energy balance observed in endurance sled dogs and begs for further understanding of not only the metabolic demands, but also appropriate feeding strategies to fuel these amazing athletes.

## Figures and Tables

**Table 1:** Dog identification, age, gender, and starting and ending body conditions for all dogs examined (*n*-12).

Dog	Age	Gender	Start	Finish
Sound	4	F	5	3
Cat	2	F	5	3
Bato	2	M	4	3
Basin	4	M	4	3.5
Heath	5	M	4	3
Yukon	5	M	5	3.5
Carbon	5	M	4	3
Braeburn	5	M	5	3.5
Chica	2	F	5	3
Krypton	3	M	4	3
Copper	5	M	5	3
Merc	5	M	5	3

## Abbreviations

IGF-1: Insulin-like growth factor-1
IGFBP-1: Insulin-like growth factor binding protein 1
IGFBP-3: Insulin-like growth factor binding protein 3
IGF-1R: Insulin-like growth factor-1 receptor.
